# Genome sequence data of *Lactiplantibacillus plantarum* IMI 507028

**DOI:** 10.1016/j.dib.2022.108190

**Published:** 2022-04-19

**Authors:** Ivana Nikodinoska, Jenny Makkonen, Daniel Blande, Colm Moran

**Affiliations:** a Alltech European Headquarters, Sarney, Summerhill Road, Co. Meath, Dunboyne, Ireland; b Biosafe – Biological Safety Solutions Ltd, Microkatu 1M, Kuopio, Finland; c Regulatory Affairs Department, Alltech SARL, Vire, France

**Keywords:** Whole genome sequencing, *Lactiplantibacillus plantarum*, Antibiotic resistance, Virulent factors, Microbial safety, Microbial identity

## Abstract

Here, we report the genome sequencing data for the fermented milk isolate, *Lactiplantibacillus plantarum* (*L. plantarum*) IMI 507028. The Bioproject, SRA, and GenBank data were deposited at NCBI under accession numbers PRJNA801616, SRR18323693, and JAKMAX000000000, respectively. The size of the genome was 3,231,321 bp, with a GC% of 44.52. Before sequence trimming, the genome contained 40 contigs, in which 35 contigs were annotated, revealing 2937 coding sequences out of 3052 total genes. The strain was identified as *L. plantarum* with an average nucleotide identity (ANI) value of 99.9922% between IMI 507028 and *L. plantarum* JDM1. Genes related to antimicrobial resistance or pathogenic factors were not found during screening.

## Specifications Table

**Table:** 

Subject	Microbiology
Specific subject area	Microbial genomics
Type of data	Raw reads, assembled and annotated data from the *L. plantarum* IMI 507028 genome sequencing
How the data were acquired	Illumina NovaSeq 6000, Unicycler v 0.4.8, PGAP v6.0, NCBI Bacterial Antimicrobial Resistance Reference Gene Database v. 2021-06-01.1, ResFinder, Virulence Factor Database (VFDB), PlasmidFinder.
Data format	Raw Analyzed
Description of data collection	*Lactiplantibacillus plantarum* IMI 507028 was isolated from fermented milk and the extracted DNA was sequenced using NovaSeq 6000 Platform (Illumina). The obtained sequencing data were used for genome analysis, strain identification, and search of genes of concern.
Data source location	Institution: Alltech Inc. City/Town/Region: Nicholasville, Kentucky Country: USA
Data accessibility	Bioproject Accession Number: PRJNA801616 NCBI GenBank Accession Number: JAKMAX000000000 NCBI SRA Accession Number: SRR18323693 Direct URL to data: https://www.ncbi.nlm.nih.gov/bioproject/PRJNA801616

## Value of the Data

• *Lactiplantibacillus plantarum* is widely isolated from fermented foods and plant sources, and is versatile for its use in products aimed at food and feed preservation.
• *Lactiplantibacillus plantarum* IMI 507028 is a strain that can find applications in the agri-food sector; therefore, its characterization is important in terms of identity and safety.
• The genome data presented herein could enrich safety-related knowledge of the lactic acid bacteria, that could be used for comparative purposes.

## 1 Data Description

*Lactiplantibacillus plantarum* (*L. plantarum*) IMI 507028 genome data, taxonomic identification, genome searches for antimicrobial resistance, and virulence (pathogenic) factors are described.

The analyzed genome sequencing data produced an annotated assembly of 35 contigs with a sequence length of 3,230,670 bp, GC% of 44.52, N50 contig length of 366,540 bp, and genome coverage of 529x. The total number of genes after annotation was 3052 genes, of which 2937 were coding sequences, 2 were ribosomal RNAs, and 59 were transfer RNA.

Genome comparison performed with alignment-free genome distance estimation with Mash using MinHash [1] showed the best hit (low distance and high matching) for *L. plantarum* JDM1 (Table 1).

**Table 1:** Taxonomic identification of IMI 507028 used as query-ID with Mash, a fast genome distance estimation using MinHash. The search was made for the closest matching NCBI RefSeq genomes. Mash distance approximates the mutation rate. The sketches are set to include 400 hashes (derived from k-mers of size 16) selected randomly from each genome for pairwise comparison. For example, IMI 507028 and the top hit *L. plantarum* JDM1 share 398 out of the 400 hashes.

Reference ID	Mash distance	*p*-value	Matching hashes	Assembly accession
*L. plantarum* JDM1	0.0001568	0	0 398/400	GCF_000023085.1
*L. plantarum* DmCS_001	0.0101152	0	296/400	GCF_000743895.1
*L. plantarum* subsp. *plantarum* NC8	0.0101152	0	296/400	GCF_000247735.1
*L. plantarum* 2165	0.0107293	0	291/400	GCF_000466845.1
*L. plantarum* LP91	0.010854	0	290/400	GCF_000473935.1

The alignment-based calculation of ANI is the most widely used comparative measurement for overall genome relatedness indices (OGRI) between two genomes to define boundaries between species with a proposed species boundary cut-off of 95–96% [2].

The genomes sequences of the indicated species included in the orthoANI genome analyses are shown in Table 2.

**Table 2:** Sequences included in the OrthoANI analysis. Type strains are marked with (T).

Strain	Assembly Accession	Contigs	Size (bp)	GC%
*L. plantarum* JDM1	GCF_000023085.1	37	3233697	44.51
*L. plantarum* subsp. *plantarum* ATCC 14917 (T)	GCF_000143745.1	1	3197759	44.66
*L. plantarum* subsp. *plantarum* ST-III	GCF_000148815.2	9	3212261	44.48
*L. plantarum* subsp. plantarum NC8	GCF_000247735.1	2	3307936	44.48
*L. plantarum* LP91	GCF_000473935.1	10	3207224	44.56
*L. plantarum* Lp90	GCF_000731855.1	145	2925584	45.06
*L. plantarum* DmCS_001	GCF_000743895.1	33	3324076	44.32
*L. plantarum* 90sk	GCF_000830535.1	83	3194687	44.54
*L. plantarum* DSM 13273	GCF_001436855.1	47	3371458	44.26
*L. pentosus* RI-031	GCF_002751855.1	90	3439800	44.24

Data from the OrthoANI comparison [3] of IMI 507028 with closely related *Lactiplantibacillus* strains are shown in Table 3. Pairwise comparisons indicated that the IMI 507028 genome shared 99.9922% identity with the strain *L. plantarum* JDM1. The cut-off value for species identification was set at 95% [4].

**Table 3:** OrthoANI (%) calculations between IMI 507028 and selected *Lactiplantibacillus* strains.

	IMI	JDM1	ATCC 14917	ST-III	NC8	LP91	LP90	DmCS_001	90sk	DSM 13273	*L. pentosus* RI-031
**IMI 507028**	100	**99.9922**	99.1327	99.0528	99.0694	99.1929	99.0239	99.0731	99.1182	99.0465	79.9773
**JDM1**		100	99.123	99.0724	99.0945	99.1721	99.052	99.0763	99.1572	99.0937	79.8184
**ATCC 14917**			100	99.2314	99.5469	99.2829	99.2928	99.5153	99.8146	99.237	79.7437
**ST-III**				100	99.3778	99.2577	99.0639	99.3811	99.211	99.6682	79.9368
**NC8**					100	99.297	99.3151	99.9935	99.5334	99.395	79.9225
**LP91**						100	99.2903	99.2424	99.225	99.2424	79.8461
**LP90**							100	99.3436	99.2952	99.0143	79.7679
**DmCS_001**								100	99.533	99.3773	79.7947
**90sk**									100	99.2086	79.9542
**DSM 13273**										100	79.8667
***L. pentosus* RI-031**											100

To assess the antibiotic resistance genes present in the sequenced genome IMI 507028, three different databases were used, and no hits with ≥80% identity and 70% coverage were identified, with criteria and thresholds proposed by the European Food Safety Authority (EFSA) for whole genome sequencing data analysis [4]. In addition, no hits were identified for toxin and virulence factor genes potentially carried by IMI 507028 when screened against an updated virulence factor database (VFDB). The search for potential plasmids was performed using PlasmidFinder, and contig 23 showed a partial match with *L. plantarum* subsp. *plantarum* P-8 plasmid LBPp6.

## 2 Experimental Design, Materials and Methods

Detailed genome sequencing data analysis was performed as previously described [5] except for the annotation performed using NCBI Prokaryotic Genome Annotation Pipeline (PGAP) v6.0 [6]. Sequencing produced 6,141,736 raw reads and 1,854,804,272 base pairs. After trimming 5,850,376 paired reads, 1,708,275,654 base pairs were obtained.

## CRediT authorship contribution statement

**Ivana Nikodinoska:** Writing – original draft, Data curation. **Jenny Makkonen:** Methodology, Software. **Daniel Blande:** Software, Formal analysis, Writing – review & editing. **Colm Moran:** Writing – review & editing, Project administration.

## Declaration of Competing Interest

The authors declare that they have no known competing financial interests or personal relationships that could have appeared to influence the work reported in this paper.

## Data Availability

SRX14461272: WGS of Lactiplantibacillus plantarum IMI 507028 (Original data) (NCBI SRA), Lactiplantibacillus plantarum strain IMI 507028, whole genome shotgun sequencing project (Original data) (NCBI GenBank), Lactiplantibacillus plantarum IMI 507028 (Original data) (Bioproject).
